# Multimorbid outpatients: A high frequency of FP appointments and/or family difficulties, should alert FPs to the possibility of death or acute hospitalization occurring within six months; A primary care feasibility study

**DOI:** 10.1371/journal.pone.0186931

**Published:** 2017-11-02

**Authors:** Jean Yves Le Reste, Patrice Nabbe, Alice Billot Grasset, Bernard Le Floch, Pauline Grall, Jeremy Derriennic, Michele odorico, Sophie Lalande, Delphine le Goff, Marie Barais, Benoit Chiron, Heidrun Lingner, Morgane Guillou, Pierre Barraine

**Affiliations:** 1 EA 7479 SPURBO, Department of general practice, Université de Bretagne occidentale, Brest, France; 2 Allgemein Medizin Hochschule Hannover, Hannover, Germany; University of Brescia, ITALY

## Abstract

**Background:**

The European General Practitioners Research Network (EGPRN) designed and validated a comprehensive definition of multimorbidity using a systematic literature review and qualitative research throughout Europe. This definition was tested as a model to assess death or acute hospitalization in multimorbid outpatients.

**Objective:**

To assess which criteria in the EGPRN concept of multimorbidity could detect outpatients at risk of death or acute hospitalization in a primary care cohort at a 6-month follow-up and to assess whether a large scale cohort with FPs would be feasible.

**Method:**

Family Physicians included a random sample of multimorbid patients who attended appointments in their offices from July to December 2014. Inclusion criteria were those of the EGPRN definition of Multimorbidity. Exclusion criteria were patients under legal protection and those unable to complete the 2-year follow-up. Statistical analysis was undertaken with uni- and multivariate analysis at a 6-month follow-up using a combination of approaches including both automatic classification and expert decision making. A Multiple Correspondence Analysis (MCA) completed the process with a projection of illustrative variables. A logistic regression was finally performed in order to identify and quantify risk factors for decompensation.

**Results:**

19 FPs participated in the study. 96 patients were analyzed. 3 different clusters were identified. MCA showed the central function of psychosocial factors and peaceful versus conflictual relationships with relatives in all clusters. While taking into account the limit of a small cohort, age, frequency of family physician visits and extent of family difficulties were the factors which predicted death or acute hospitalization.

**Conclusion:**

A large scale cohort seems feasible in primary care. A sense of alarm should be triggered to prevent death or acute hospitalization in multimorbid older outpatients who have frequent family physician visits and who experience family difficulties.

## Introduction

The concept of multimorbidity was first published in 1976 [1]. Multimorbidity has been defined by the World Health Organization (WHO) as people being affected by two or more chronic health conditions [2]. The word ‘condition’ was not sufficiently clear for practical purposes (for instance, whether a treated disease was a ‘condition’ in this sense), and led to numerous interpretations. Many authors tried to achieve a consensus on a definition for multimorbidity and to disentangle the concepts of comorbidity, multi-pathology and multimorbidity without reaching a formal consensus [3] [4]. This was especially complicated when using patient databases to assess what could be encompassed by multimorbidity [5][6]. Due to that lack of systematization, it remained difficult to determine which outcomes could be predicted for patients[7] and how the management should be improved in a primary care setting [8].

Nevertheless, multimorbidity remained a very interesting and challenging concept particularly for Family Medicine, given the increasing prevalence of chronic illness in an aging population across all developed countries. It appeared closely related to a global or comprehensive view of the patient, which is a core competency of Family Medicine (FM), as defined, for instance, by the World Organization of National Colleges, Academies and Academic Associations of General Practitioners/Family Physicians (WONCA) [9]. It is definitely a global ‘functional’ view (useful for Long-Term Care) versus a ‘disease’ centered point of view (useful for acute care) [10].

The European General Practice Research Network (EGPRN), being committed to concepts that could advance research in primary care throughout Europe, was interested in multimorbidity when it created its research agenda [11]. A comprehensive definition of the concept of multimorbidity (i.e. one which is both understandable and usable for further collaborative research) was an important objective for a research network of this type. It could help researchers in FM to investigate the complexity of patients’ conditions and their overall impact on patients’ health. This concept of multimorbidity could be an additional tool for Family Physicians (FPs), enabling them to identify frail patients and, eventually, prevent severe outcomes like death or hospitalization in acute wards. [12].

A research team, including 9 national groups, all active within the EGPRN, has created a research community for the purpose of clarifying the concept of multimorbidity for FM throughout Europe [13]. This group produced a comprehensive definition of the concept of multimorbidity through a systematic review of literature [14]. Even though this definition does not include the thoughts and reactions of major authors on the concept [4], it was the first attempt within Europe to reach a consensus validated by practicing FPs using a pragmatic methodology[15]. This definition was translated into 10 European languages for use in further collaborative research [16]. The translations included (in addition to English) most major European countries, in terms of population size, such as France, Germany, Italy, Poland, Spain and some less populated countries, such as Bosnia, Bulgaria, Croatia and Greece and the region of Catalonia in northern Spain. The interest shown by these FPs indicated the importance of the concept throughout Europe, for countries with a high GNP as well as those with a lower GNP, for established and newly democratic states and for all types of language (from Romance and Germanic to Slavic). A specific research agenda was finally issued [12] which led to the need for surveys to validate quantitatively and to simplify the concept.

The EGPRN concept of multimorbidity is defined as any combination of chronic disease with at least one other disease (acute or chronic) or bio-psychosocial factor (associated or not) or somatic risk factor. Any bio-psychosocial factor, any somatic risk factor, the social network, the burden of diseases, the health care consumption, the patient’s coping strategies, the FP’s expertise and the doctor-patient relationship dynamics may function as modifiers (of the effects of multimorbidity). Multimorbidity may modify the health outcomes and lead to an increased disability or a decreased quality of life or frailty. Nevertheless this comprehensive definition, as others, leads to a major pitfall, which is precisely due to its comprehensive nature, in that it has the potential to include almost all patients in a primary care setting, as nearly everyone might appear to be multimorbid…

To try to simplify the concept, the EGPRN research team chose to use its outcomes to find which variable could be useful for the patient in preventing some severe outcomes. Detecting a risk of death or of acute hospitalization remains a challenge for FM, as well as for FPs who, being familiar with their patient’s health status [17], could miss very small factors which, if noticed, could help to prevent these two severe outcomes [18]. The research group assumed that death or unplanned hospitalization in acute care would provide a useful model for severe outcomes [19]. Such a predictive model that could be integrated, for example, into their professional software could help FPs to prevent these two severe outcomes [20].

The purpose of this research was to assess which of the FPs’ criteria, within the EGPRN concept of multimorbidity, could detect multimorbid outpatients at risk of death or acute hospitalization in a primary care cohort in France at a 6-month follow up and which would also function as a feasibility study for designing a European-wide study.

## Material and methods

The survey was a prospective cohort study to derive a risk score and strictly follows the TRIPOD statement [21] for a development-only survey as just a single data set was available and all data was used to develop the model.

### Ethics statement

The study was approved by the ethics committee of the University de Bretagne Occidentale. The participants (FPs and patients) provided their written informed consent to participate in the study. The ethics committee approved the consent procedure.

### Research team

Two senior FM researchers, one statistician and 2 trainees in FM constituted the research group to improve participant selection and to support the FPs in the inclusion and follow up procedure. A scientific committee composed of 8 senior researchers in primary care (from 6 European countries) supervised the survey.

### Participant selection

The study population included a random sample of multimorbid patients (according to the EGPRN definition of multimorbidity). Patients were selected by 19 FPs in their offices in the county of Finistere (in north-west France) from July 2014 to December 2014. These FPs were drawn from the Clinical Teachers list of Brest University. Randomization was achieved by including the first four multimorbid patients (according to the inclusion criteria) encountered during their second working day of each week. As patients were booking their appointments with the practice without the FPs’ clearance, the FPs were not able to select them.

Inclusion criteria were patients meeting the criteria for the definition of multimorbidity according to the EGPRN definition: any combination of chronic disease with at least one other disease (acute or chronic) or a bio psychosocial factor (associated or not) or a somatic risk factor.

Bio-psychosocial factors meant all psychosocial risk factors, such as lifestyle (for example: healthy diet or otherwise, physical activity or otherwise, social isolation), psychological distress, socio-demographic characteristics, aging, beliefs and expectations of patients.

In all cases, monitoring over time was required and the patient had to sign an informed consent.

Exclusion criteria were patients not meeting the criteria of the definition of multimorbidity, the inability to follow the study over time (known to be leaving the practice in the following months), patients under legal protection, outpatients in palliative care for whom survival was estimated by the FPs at less than three months.

### Data collection

From July 2014 to December 2014, FPs who had agreed to participate worked according to the following plan: first, the multimorbid patient was asked to give his/her consent to participate in the study once the terms had been explained to him/her. After that, FPs completed a paper questionnaire about their patient.

This questionnaire was to explore potential decompensation risk factors within themes and subthemes of multimorbidity (Table 1)

**Table 1:** Themes and type of related variables identified for multimorbidity conditions. All variables were reported by physicians and then double-checked in medical files by the research team with the exception of those which reported FPs’ feelings.

Themes	Subthemes	Variable type	Answer type
Chronic disease	Chronic diseases	Quali (type) & quanti (number of)	Count and Check in a list based on ICD 10
	Complexity characteristics of chronic disease	Quali	Yes/No referring to FP’s feeling about the patient
	Psychosomatic diseases	Quali	Yes/No referring to a fixed list
Acute disease	Acute condition	Quali	Yes/No referring to a fixed list
	Acute disease	Quali	Yes/No referring to a fixed list
	Complexity characteristics of acute disease	Quali	Yes/No referring to FP’s feeling about the patient
	Reaction to severe stress and acute disorders	Quali	Yes/No referring to FP’s feeling about the patient
Biopsychosocial factors and somatic risk factors	Demography risk factor	Quali	Yes/No referring to a fixed list
	Lifestyle: healthy diet and hygiene	Quali	Yes/No
	Psychological risk factors	Quali	Yes/No referring to a fixed list about suicide risk, addiction,
	Psychosocial risk factors	Quali	Yes/No referring to a fixed list about marital problems, family problems, death of relatives
	Sociodemographic characteristics	Quali (Gender) & quanti (age)	Female/male and age
	Somatic risk factors	Quali	Yes/No referring to a fixed list including hypercholesterolemia, hypertension, excess weight, immunosuppression,
Coping	Patient’s coping strategies	Quali	Yes/No referring to a fixed list
Burden of diseases	Extensive and complex medical History	Quali	Yes/No referring to FP’s feeling about the patient
	Chronic disease complication including iatrogeny	Quali	Yes/No referring to medical files
Health care consumption	Use of carers	Quanti	Count of medical and paramedical interventions per year
	Disease management	Quali	Yes/No referring to a fixed list regarding therapeutic educational equipment, FP negligence, lack of time and remuneration, human help and coordination between carers
	Prevention: Health system screening and immunization recommendations	Quali	Yes/No according to medical files
	Health system screening acceptance by patient	Quali	Yes/No according to medical files
	Health care services use	Quanti	Count of biochemical and imaging procedures per year
	number of medications per day	Quanti	Count of medications in medical files
	Communication needed between carers	Quali	Yes/No referring to FP’s feeling about care
	Pain	Quali	Yes/No referring to FP’s feeling about the patient
	Treatment with risks and daily use of psychotropics	Quali	Yes/No referring to FP’s feeling about the patient
Disability	Handicap	Quali	
	Functional impairments: postural instability	Quali	CETAF score for Falls risk
Social network	Dependency on entourage	Quali	Yes/No referring to FP’s feeling about the patient
	Entourage has some coping strategies	Quali	Yes/No referring to FP’s feeling about the patient’s entourage
	Presence of entourage	Quali	Yes/No referring to FP’s feeling about the patient
	Supportive entourage	Quali	Yes/No referring to FP’s feeling about the patient
Health outcomes	Decompensation	Quali	Yes/No and dates
	Mortality	Quali	Yes/No
Core competencies of FP	Health system management knowledge	Quali	Yes/No referring to FP’s feeling about his/her care
	Long term relationship usefulness in multimorbid care	Quali	Yes/No referring to FP’s feeling about his/her care
	FP’s habits of complex problem solving skills	Quali	Yes/No referring to FP’s feeling about his/her care
	"gut feeling"/intuition usefulness in multimorbid care	Quali	Yes/No referring to FP’s feeling about his/her care
	Person-centered care usefulness in multimorbid care	Quali	Yes/No referring to FP’s feeling about his/her care
	Global overview of diseases: its usefulness in multimorbid care (Holistic Approach)	Quali	Yes/No referring to FP’s feeling about his/her care
Relationship between FP and patient	Quality of communication between patients and FPs	Quali	Yes/No referring to FP’s feeling about his/her patient
	Influence of Multimorbidity on quality of follow up	Quali	Yes/No referring to FP’s feeling about his/the patient

Quali for qualitative variable, Quanti for quantitative variable.

The research group developed this questionnaire according to the definition of multimorbidity. Some subthemes in multimorbidity definitions were impossible to assess (as explained below) and were discarded to obtain the list of variables used in Table 2. To evaluate the concept of somatic risk factors, the team retained variables around a cardiovascular risk factor, a risk of falling factor, an assessment of hygiene, nutrition and physical activity. The risk of falling was calculated using the CETAF score [22]. A first pilot study was used to delete some irrelevant variables: chronic condition (previously described as being linked to chronic disease or psychological risk factor), cost of care (impossible to estimate given the time and resources dedicated to the study), disability (disability / impairment), quality of life and health outcome (which are the consequences, rather than the characteristics, of multimorbidity), demography and aging (already assessed in sociodemographic characteristics). This questionnaire was accepted by the scientific committee of the research team and tested with FPs and medical students. It was formatted to conform to computerized data collection with the help of EVALANDGO ® software (online survey software). Data were saved using Microsoft Excel. Six months after inclusion (January to June 2015), FPs were contacted by email to check patient status. The collected data were anonymized and a number was assigned to each patient, in order of inclusion, for statistical analysis. The patient was then classified into either group according his/her status: decompensation (D) or nothing to report (NTR). The team defined decompensation, in this context, as the occurrence of hospitalization for more than six days or death during the six months of the follow-up. As the mean duration for hospitalization in the European Union is 6.7 days, it was used as a cut-off between acute hospitalization for less severe conditions (allowing a quick release) and severe cases[23] The research team called the FPs to gather these data and searched the electronic records of the practices.

**Table 2:** Characteristics of D group and NTR group for each variable as described by the FPs.

	Study population N = 96	Decompensation (D) N = 13 (13.5%)	Noting to report (NTR) N = 83 (86.5%)	p.value (Rounded)
Men	48.96%	53.85%	48.19%	1
Women	51.04%	46 .15%	51.81%	1
**Average age**	**70.85**	**78.54**	**69.65**	**0.044**
farmer	11 (11.46%)	1 (7.69%)	10 (12%)	0.641
skilled manual worker	10 (10.42%)	2(15.38%)	8 (9.64%)	0.641
manager	5 (5.21%)	1 (7.69%)	4 (4.82%)	0.641
employed	10 (10.42%)	1 (7.69%)	9 (10.84%)	0.641
Semi-skilled/unskilled worker	16 (16.67%)	4 (30.76%)	12 (14.46%)	0.641
Intermediate professional	15 (15.62%)	2 (15.38%)	13 (15.66%)	0.641
unemployed	29 (30.21%)	2 (15.38%)	27(32.53%)	0.641
Single or widowed	52 (81.25%)	9 (75%)	43 (82.7%)	0.641
Number of chronic diseases	4.083	4.769	3.976	0.125
Hypertension	55 (57.29%)	8 (61.54%)	47 (56.63%)	1
Hypercholesterolemia	35 (36.46%)	3 (23.08%)	32 (38.55%)	0.363
Diabetes	27 (28.13%)	3 (23.08%)	24 (28.92%)	1
Osteoarticular disease	55 (57.29%)	9 (69.23%)	46 (55.42%)	0.386
Psychosomatic disease	37 (38.54%)	6 (46.15%)	31 (37.35%)	0.555
Complexity of chronic disease	52 (54.17%)	7 (53.85%)	45 (54.21%)	1
Acute disease	0.653	0.779	0.639	0.469
Reaction to severe stress	32 (33.33%)	7 (53.85%)	25 (30.12%)	0.117
Complexity of acute disease	9 (9.38%)	3 (23.08%)	6 (7.23%)	0.101
Excess weight	32 (33.33%)	4 (30.77%)	28 (33.73%)	1
Immunosuppression	12 (12.5%)	2 (15.38%)	10 (12.04%)	0.664
Postural instability (CETAF score)	52 (54.17%)	10 (76.92%)	42 (50.60%)	0.132
Average number of falls in a year	0.385	0.769	0.3854	0.078
Suicide risk	4 (4.17%)	2 (15.38%)	2 (2.4%)	0.087
Addiction	14 (14.58%)	1 (7.70%)	13 (15.66%)	0.684
Marital problems	8 (8.33%)	2 (15.38%)	7.23 (2%)	0.296
**Family problems**	**13 (13.54%)**	**6 (46.15%)**	**7 (8.43%)**	**0.002**
Death of one or more relatives	11 (11.46%)	1 (7.69%)	10 (12.05%)	1
Good hygiene	71 (73.96%)	10 (76.9%)	61 (73.49%)	1
Physical activity	35 (36.46%)	7 (53.85%)	28 (33.73%)	1
Healthy diet	46 (47.91%)	9 (69.23%)	37 (44.58%)	0.137
Children	11 (11.46%)	1 (7.69%)	10 (12.05%)	1
Coping strategy	62 (64.58%)	8 (61.54%)	54 (65.06%)	1
Complication of chronic disease	32 (33.33 %)	7 (53.85%)	25 (30.12%)	0.117
**Number of FP consultations per year**	**10.67**	**18.77**	**9.40**	0.009 *10^**−9**^
**Number of specialist consultations per year**	**4.229**	**6.769**	**3.831**	0.002 *10^**−7**^
Number of times health paramedics used per year	89.15	127.6	83.12	0.438
Treatment with risks	34 (35.42%)	6 (46.15%)	28 (33.73%)	0.534
Daily use of psychotropics	45 (46.88%)	6 (46.15%)	39 (46.99%)	1
Coordination procedure	50 (52.08%)	7 (53.85%)	43 (51.81%)	1
Number of biochemical tests per year	2.156	3	2.024	0.015
Number of medical imaging procedures per year	1.354	1.308	1.361	0.124
Inadequate communication between carers	88 (91.67%)	12 (92.31%)	76 (91.57%)	0.296
FP Negligence towards patient	4 (4.17%)	**0 (0%)**	4 (4.81%)	1
¨Human help at home	37 (38.54%)	**7 (53.85%)**	34 (32.53%)	0.238
**Equipment for patient at home**	**21 (21.86%)**	**6 (46.15%)**	**15 (18.07%)**	**0.033**
Patient victim of iatrogeny	13 (13.54%)	1 (7.69%)	12 (14.46%)	1
**Lack of time or remuneration**	**21 (21.88%)**	**6 (46.15%)**	**15 (18.07%)**	**0.033**
**Extensive and complex medical history**	**73 (76.04%)**	**13 (100%)**	**60 (72.29%)**	**0.034**
Vaccination recommended	73 (76.04%)	12 (92.30%)	61 (73.49%)	0.179
Screening proposed	65 (67.70%)	5 (38.47%)	60 (72.29%)	0.237
Screening accepted	65 (95.59%)	10 (76.92%)	55 (66.26%)	0.538
Therapeutic education offered	21 (21.86%)	1 (7.69%)	20 (24.10%)	0.286
Pain	49 (51.04%)	6 (46.15%)	43 (51.80%)	0.772
Multiple complaints	31 (32.29%)	6 (46.15%)	25 (30.12%)	0.339
Number of medicinal treatments	7.302	8.462	7.120	0.873
Presence of entourage	79 (82.29%)	12 (92.31%)	72 (86.75%)	1
Supportive entourage	57 (59.38%)	8 (61.51%)	49 (59.03%)	1
Dependency on entourage	18 (18.75%)	5 (38.46%)	13 (15.66%)	0.064
Entourage able to cope	16 (16.67%)	4 (30.77%)	12 (14.46%)	0.221
Heath system management knowledge	83 (86.46%)	11 (84.62%)	72 (86.75%)	1
Skills for complex problems solving	92 (95.83%)	12 (92.30%)	80 (96.39%)	0.446
Global overview of diseases	95 (98.96%)	12 (92.30%)	83 (100%)	0.135
Person centered care	94 (97.91%)	12 (92.30%)	82 (98.80%)	0.254
Long term relationship	95 (98.96%)	12 (92.30%)	83 (100%)	0.135
Intuition	67 (69.79%)	11 (84.61%)	56 (67.47%)	0.332
Quality communication	95 (98.96%)	12 (92.30%)	83 (100%)	0.135
Multimorbidity influence on quality of follow up	77 (80.20%)	12 (92.30%)	65 (67.70%)	0.454

Significant p value lines are in bold. P values are rounded to the nearest thousandth (equal to or more than 0.0005 rounded to 0.001, less than 0.0005 to 0.000)

### Data analysis

Data cleaning was performed to harmonize the data before statistical analysis. Descriptive statistics were used to check data quality and consistency. During this step, missing data were identified. For example, some questions about the number of biological analyses and the number of scans performed each year, apart from monitoring, were unanswered in the case of a few included patients. These missing data had to be identified and to be included in the statistical analysis (missing data were replaced by the median value of the group). The most important recoding work took place during data cleaning: the number of acute and chronic diseases was recalculated by FP trainees from free text fields completed by FP recruiters. 102 chronic diseases were taken into account. Every change and its rationale was recorded in “a dictionary” which is available on demand from the corresponding author.

2 steps were used for the analysis. These two steps are not directly connected but they both relate to the study question. Step 1 was used to describe the population of multimorbid patients. A Multiple Correspondence Analysis (MCA) projected the initial variables onto a scaled space that was used to cluster the patients. The methods resulted in 3 clusters, which highlighted relevant characteristics. This multidimensional and descriptive approach made it possible to follow sub-groups of patients separately, based on these clusters. Then, step 2 pursued a different objective, as the status at 6 months is was a variable to be explained within a regression procedure. These 2 steps are carefully described below.

Uni- and two-dimensional analyses for qualitative variables were performed. Fisher's exact test was performed for each categorical variable. Fisher-Snedecor procedures (i.e. F test) were used to compare two population sub-groups where needed. Multidimensional analyses were then combined to bring together individuals with common characteristics in groups and thus describe the population of multimorbid patients, regardless of their status at six months. An MCA was first performed as a factorial method able to reduce the dimensions. This technique is suitable for qualitative variables only. It consisted of a representation of the individuals (the patients) in a factorial space where each dimension is a combination of the initial variables. Quantitative variables are not used during the procedure but they are then projected, *a posteriori*, onto the factorial space. Based on the individual’s coordinates, the technique of Hierarchical Clustering on Principal Components (HCPC) was then performed in order to highlight groups of patients sharing common characteristics. The combination of MCA and HCPC helped in the interpretation of the resulting groups. In order to evaluate the optimal number of groups in the dataset (a classic issue in clustering) and check the clustering stability, an alternative procedure was then carried out. A dissimilarity measure, suitable for mixed data, was selected (the Gower distance) and both k-means and hierarchical clustering (with the Ward method) were launched. The same clustering structure was obtained. The use of a clustering quality index silhouette, as described by Kaufman, was performed[24]. Each cluster was then defined and interpreted by a list of qualitative and quantitative variables for which the proportion, or mean of the group, was interpreted in comparison with the global rate or the overall mean.

Finally a logistic regression was undertaken with the objective of predicting and explaining the patient's status at 6 months. In the case of the general linear model, logistic regression is a suitable tool because the dependent variable is binary: D (decompensation) versus (vs) NTR (Nothing to Report). The goal is to find the best subset of variables able to explain the decompensation. In this way, a mixed variable selection procedure, backward-forward, was applied with Akaike information criterion (AIC). The shortcomings of this procedure were minimized by combining the results with expert knowledge. The Wald significance tests were used to strengthen the proposed interpretations and the relevance of the results.

## Results

### Sample participants

102 patients were included by 19 FPs. Out of the 102 patients, only 96 were analyzed, 5 were excluded because of incomplete questionnaires or because a questionnaire was filled in twice for the same patient. The status at 6 months was collected for all patients. None was lost in follow up. Each physician included between 8 and 13 patients. All the clusters found below were represented according to each physician’s recruitment.

### Data cleaning and recoding

The “divorce” variable was removed from the analysis because all answers were “no”. The recoding data work was transcribed in a dictionary (available on demand from the corresponding author).

### Characteristics of the included patients

**Fig 1:**
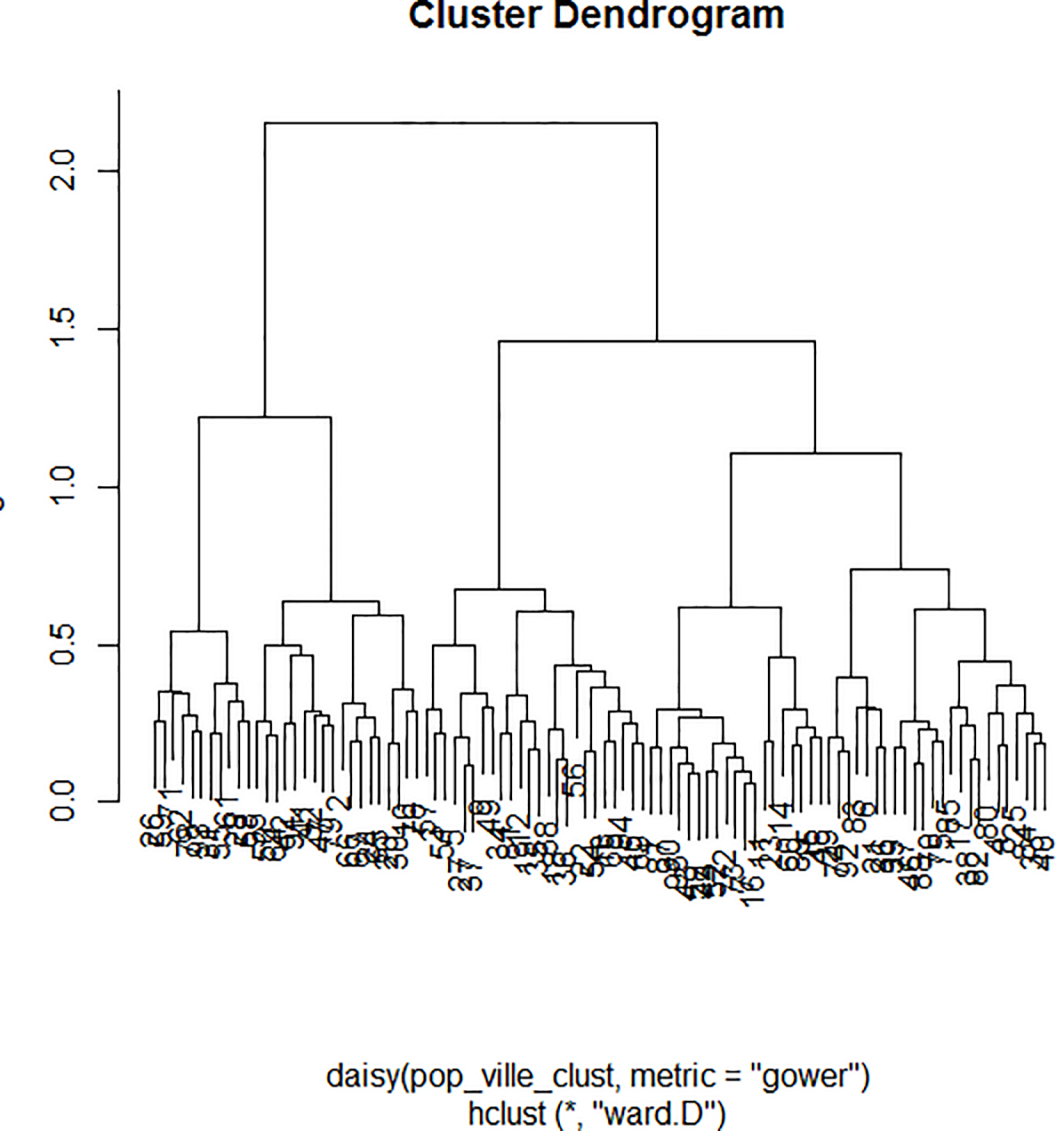
Cluster dendrogram.

**Fig 2:**
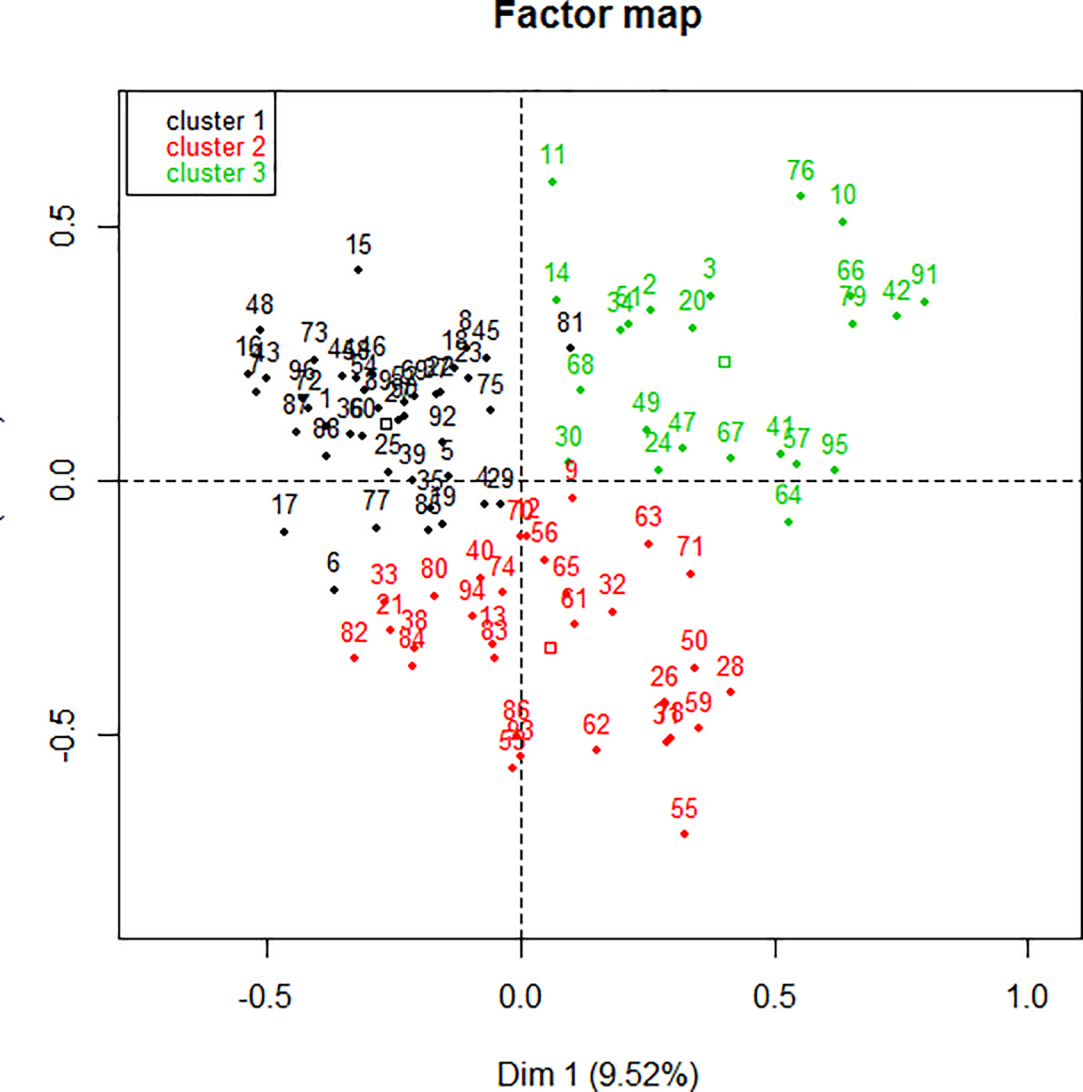
Factor map.

The dendrogram (Fig 1) allowed observation of the hierarchical groups formed from the aggregation of individuals during the hierarchical clustering. The height of a branch is proportionate to the distance between individuals or groups of individuals. Graphically, the dendrogram suggested between 3 and 5 groups whereas a clustering quality index was maximized for 3 groups of patients. After discussion among the group of experts, the model with 3 clusters was also the most meaningful from a clinical perspective. Fig 2 depicts the 3 groups of individuals projected onto the MCA factor map (first two dimensions).

In order to interpret each patient group, a comparison of the proportion within the group (PwG) and within the study population (PwP), that is the 96 patients, allowed the team to understand the importance of a variable as a measure of specific characteristics, in order to determine a group of patients. For quantitative variables, a comparison of the mean within the group (MwG) and within the study population (MwP) needed to be finalized.

#### Cluster 1

This was the largest cluster with 43 patients. All had good hygiene (PwG: 100% vs. PwP:74%), none had a psychological risk factor (PwG: 100% vs. PwP:77%) none had a particular lifestyle factor (PwG: 100% vs. PwP:81%), and none showed addictive behavior (PwG: 100% vs. PwP:85%). Most had regular physical activity (PwG: 67% vs. PwP:36%), healthy eating habits (PwG: 71% vs. PwP:48%) and supportive relatives (PwG: 73.8% vs. PwP:59.4%). Often, patients were couples (PwG: 71% vs. PwP:58%). A large majority had little complexity within their chronic diseases (PwG: 67% vs. PwP:46%) but were not multiplaintive (PwG: 81% vs. PwP:68%).This cluster was defined by a lower number of acute pathologies (MwG: 0.5 vs. MwP:0.6, p<0.05), a smaller CETAF score (MwG: 3 vs. MwP: 4, p<0.05) and lower use of paramedics (MwG: 9 vs. MwP:89, p<0.05) than the population’s mean.

#### Cluster 2

This group contained 29 patients. All patients in this group had two psychosocial risk factors: stress due to occupation (PwG: 100% vs. PwP:92%) and relationship issues with their partner (PwG: 100% vs. PwP:92%). A large majority had postural instability (PwG: 77% vs. PwP:54%), human help (PwG: 84% vs. PwP:38%) and medical equipment available at home (PwG: 58% vs. PwP:22%). In most of cases, patients in this group lacked physical activity (PwG: 87% vs. PwP:63%) and lacked hygiene (PwG: 48% vs. PwP:26%). Frequently, patients were single (PwG: 61% vs. PwP:42%) with at least one dependent relative who did not live with them (PwG: 48% vs. PwP:19%) and had developed adaptative strategies to cope with this situation (PwG: 39% vs. PwP:17%). This group was associated with greater use of paramedics (MwG: 253 vs. MwP: 89, p<0.05), a higher age (MwG: 78 vs. MwP:71, p<0.05) and a greater number of chronic diseases (MwG: 4.8 vs. MwP: 4, p<0.05) than the population’s mean.

#### Cluster 3

Twenty three patients were classified in this group. People with an addictive behavior (PwG: 61% vs. PwP:15%) and psychosomatic disease (PwG: 74% vs. PwP:38%) were over represented. In this context, an important proportion experienced marital problems (PwG: 30% vs. PwP: 8%), financial and social insecurity (PwG: 43% vs. PwP: 19%). An unhealthy diet (PwG: 78% vs. PwP: 52%), excess weight (PwG: 56% vs. PwP: 33%) and failure to comply with screening (PwG: 52% vs. PwP: 32%) were frequently combined. Usually the FPs managed to propose therapeutic education (PwG: 43% vs. PwP: 22%) for which they may have felt they lacked time (PwG: 39% vs. PwP: 22%). Manual workers were often found in this group (PwG: 39% vs. PwP: 16%). Most of the group did not attempt to adapt by using any strategies at all in order to feel better (PwG: 56% vs. PwP: 35%) and three times as many patients in this group had attempted suicide (PwG: 13% vs. PwP: 4%). Patients were younger in this group (MwG: 59 vs. MwP:71, p<0.05) and had used paramedics less often (MwG: 14 vs. MwP: 89, p<0.05) than the population’s mean.

### Status at 6 months

Six months after their inclusion, the population was divided into two groups according to their status. 13 patients belonged to the “Decompensation” (D) group and 83 belonged to the “Nothing to report” (NTR) group. Among the D group, 10 patients had been hospitalized for more than six days and 3 had died. The characteristics of each group are reported in Table 2.

Several variables compared were significant (p < 0.05):

For the patients in the “Decompensation group,” the following applied:

- They had more family problems (46.15% versus 8.43%; p-value = 0.002)
- The presence of equipment was significantly higher (46.15% versus 18.07%; p-value = 0.033).
- The impression of lack of time and remuneration was higher (46, 15% versus 18, 07%; p-value = 0, 033).
- The medical history was more extensive and more complex (100% versus 72.29 p-value = 0.034).

A test of comparison of variance was undertaken for quantitative variables in order to interpret differences observed in box plots.

For the patients in the “Decompensation group,” the following applied:

- Were significantly older than those in the “NTR” group: average age of 78.54 years in the decompensation group compared with 69.65 years in the NTR group (p -value = 0.044).
- Had an average annual use of biochemical testing, other than for monitoring purposes, which was higher than in the NTR group (3.31 compared with 2.28, p-value = 0.015).
- The number of consultations with a general practitioner was significantly higher than in the “NTR” group: (18.77 per year compared with 9.40 per year; p-value = 0.009*10^−9^).
- The number of visits to a specialist was lower than in the “NTR” group (3,8versus 6,8 p-value = 0.002*10^−7^).

### Logistic regression

The initial model included all variables with the removal of non-discriminatory variables. An automatic selection held 16 variables (available on demand from the corresponding author) with substantial "standard error". It was necessary to integrate some expert knowledge at this level to reduce the number of variables. Some Chronic diseases and their names were removed (osteoarticular diseases, diabetes, high cholesterol, high blood pressure) because the *number* of chronic diseases was preferable to their *type* (for which the list was not exhaustive). In Table 3, the final model, composed of the most significant variables and associated confounding factors (not necessarily significant) is presented. As family problems was a significant factor, the research group wished to check up on marital status, supporting entourage and dependency on entourage as possible co-factors of family problems leading to the regression model described in Table 3.

**Table 3:** Final regression model.

	Coeff	OR	p.value
Intercept	-20.3335	0.00	0.003
**Age**	**0.1741**	**1.19**	**0.013**
Couple	1.8787	6.54	0.094
Supporting entourage	0.2603	1.30	0.788
Dependency on entourage	-0.1988	0.82	0.849
complexity of chronic disease	-0.6634	0.52	0.540
Psychosomatic disease	1.3524	3.87	0.281
**Family problems**	**2.0731**	**7.95**	**0.055**
**Number of visits to FP**	**0.2639**	**1.30**	**0.008**
Patient in pain	1.5417	4.67	0.162

Significant p value lines are in bold. P values are rounded to the nearest thousandth (equal to or more than 0.0005 rounded to 0.001)

Age was a risk factor for decompensation (OR 1.19; p-value = 0.013).

The number of visits to FP per year was significantly associated with a decompensation outcome (0R 1.30; p-value = 0.008). Family problems were linked to decompensation (0R 7.95; p-value = 0.055) but not significant on their own.

### Key points

57% of patients who had made more than 17 visits to their FP per year had decompensated during the following 6 months (p<0.001).

45% of patients with family problems and who had made fewer than 17 visits to their FP per year had decompensated within 6 months (P< 0.001).

5% of patients who had made fewer than 17 visits to their FP per year and had no family problems had decompensated within 6 months.

## Discussion

### Main results

The survey seems feasible on a larger scale, as FPs did not encounter difficulties in including multimorbid outpatients, in completing the questionnaire or in following up patients’ status at six months. Among the themes of the definition of Multimorbidity, 3 variables emerged to explain decompensation: « age », « number of visits to FPs» and « family problems». Age was important as it was already known that age alters multimorbidity and this result showed the consistency of the feasibility study [25]. The number of visits to FPs and the presence of family problems were the two additional and useful variables that could help to prevent decompensation.

### Strengths and limitations of the study

#### Selection bias

Recruiting FPs knew the study endpoint and the aim of the study. Knowing these criteria, FPs could have recruited those patients most at risk of decompensation [26]. This effect was reduced by one of the exclusion criteria « the estimated survival lower than three months » and by the need to recruit the first four patients on the FP’s second consultation day each week.

#### Information bias

The missing data were taken into account. The team imputed the median of the group for statistical analysis to reduce information bias.

*The cluster of chronic disease* to be used was decided by the scientific committee. A wide range of diseases was selected (102 chronic diseases). In literature, a list of at least 12 chronic diseases was recommended because variations in the prevalence results were less significant beyond a total of twelve diseases [27].

*The CETAF score* was not validated for patients under 65 years of age. The team assumed that this score would not be high enough below 65 years of age and would not change the statistical results.[28]

Finally, readers should carefully take into account that most data were collected by the FPs athough some additions and checking required reference to the medical files. This could lead, to an information bias. Nevertheless, this is exactly what the study was designed for. The scientific committee of the study assumed that it would be interesting to look into FPs’ feelings about their patients in a feasibility study.

#### Confounding bias

This study was performed using the most appropriate statistical method. However, the small number of patients compared to the large number of variables was the source of many difficulties throughout the analysis. Some choices were made to reduce the number of variables: variables that were not statistically relevant were removed from the analysis.

Finally, the judgment criterion chosen for this study was hospitalization for more than six days or death. This choice avoided a confounding bias because it was objective, clinical, easily measured and suited the question. Nevertheless, it remains debatable.

### Key points

Age, the number of visits to the FP and family problems were the three variables that summarized the information found useful for predicting decompensation at 6 months.

Many studies have been conducted to assess the relationship between multimorbidity and health outcomes but without solving the problem of the meaning or the intensity of that relationship [29,30]. The most effective variables identified by this study simplify the concept when the specific outcome of decompensation is targeted. This is helpful for clinicians in everyday practice. At the same time, it could help to resolve the debate around measuring multimorbidity as FPs are able to simplify a concept which academics have found to be broad and complex.

Focusing on the number and description of all active chronic diseases seemed pointless. Much of the multimorbidity prevalence research used a list of chronic diseases[31][32][33]. However, those lists of chronic diseases were very different. In addition, the criteria for the selection of the diseases were often pragmatic[34]. Based on this observation, the team chose to consider almost all chronic diseases listed by the FPs. However, in the end, this was not important for predicting decompensation. Much research has targeted clusters of diseases which now seems infeasible for predicting decompensation in pragmatic care, even though a more statistically powerful study may be needed to confirm this result.

### Implications for practice, teaching and future research

In practice, age, number of FP visits and family problems should alert FPs to the possibility of decompensation in a multimorbid outpatient within the following 6 months. These variables are easy to monitor in primary care and seem pragmatic in family practice. This result should, nevertheless, be used cautiously as too many biases would make the study worthless.

In teaching activities, trainees in FM should be made aware of the importance of these “risk or alarm factors”. The same restrictions apply here as apply in family practice.

In future research, these results in a small sample, in only one country, should be confirmed by a large scale European study. This study is included in an EGPRN project which aims to define the best possible intervention in order to prevent decompensation in multimorbid patients within 11 European countries. Due to the intentional information bias of the study (focusing on FPs’ feelings about their patients) some variables should be drawn from patients in future studies. For example hygiene, physical activity… Nevertheless, finding results with a small sample is encouraging the research team to follow the hypothesis that the outcomes of multimorbidity could help to simplify the concept.

## Conclusion

The number of visits to FPs, family problems and age seem to be important in multimorbid outpatient decompensation. The number of chronic diseases and the burden of these diseases did not make any difference. A large scale study seems feasible for assessing whether the prevention of decompensation, through the use of the EGPRN concept of Multimorbidity, could make research and everyday practice in primary care more straightforward.
